# Possibilities of Obtaining from Highly Polluted Environments: New Bacterial Strains with a Significant Decolorization Potential of Different Synthetic Dyes

**DOI:** 10.1007/s11270-018-3829-7

**Published:** 2018-05-21

**Authors:** Ewa Zabłocka-Godlewska, Wioletta Przystaś, Elżbieta Grabińska-Sota

**Affiliations:** 1 Faculty of Energy and Environmental Engineering, The Silesian University of Technology, Akademicka 2A, 44-100 Gliwice, Poland; 2 Biotechnology Center, The Silesian University of Technology, B. Krzywoustego 8, 44-100 Gliwice, Poland

**Keywords:** Decolorization, Wastewater, Polluted river water, Bacteria, Synthetic dyes, Azo dyes, Triphenylmethane dyes, Fluorone dyes

## Abstract

The aim of this study was the isolation of bacterial strains which have the ability to decolorize synthetic dyes belonging to different chemical groups. The samples for bacterial isolation were collected from aqueous environments—two activated sludges and polluted local river. At the first stage of screening (performed on the solid media supplemented with two dyes—azo Evans blue or triphenylmethane brilliant green), 67 bacterial strains were isolated capable to decolorize the used dyes. In the further study, six dyes with different chemical structures were used: fluorone dyes (Bengal rose, erythrosine), triphenylmethane dyes (brilliant green, crystal violet), azo dyes (Evans blue, Congo red). Initial concentration of each of these chemicals in samples was 0.1 g/l. Obtained results showed that only 31 isolates were able to decolorize all six used dyes (with different efficiencies). Among them, 11 strains were isolated from the river (55% of isolates from this site) and 20 from activated sludges collected from two different treatment plants (15 from the first water treatment plant and 5 from the second which were 42 and 43% of isolated cultures respectively). The decolorizing microorganisms are mostly isolated from different industrial sewages (e.g., textile industry), but results of the study showed that water from polluted river as well as municipal wastewaters may be a precious source for isolation of bacterial strains with the wide spectrum and high decolorization potential. In general, there were no statistically significant differences between decolorization abilities of strains isolated from different sites. The group of dyes that was removed with the highest yield was triphenylmethanes (75.6%), followed by fluorones (70.0%) and azo group (60.9%). The analysis of decolorization efficiency of the individual dyes revealed the best removal results in case of triphenylmethane brilliant green (average removal 85.7%), followed by fluorone erythrosine (average removal 78.9%), triphenylmethane crystal violet (average removal 65.5%), azo Evans blue (average removal 64.4%), fluorone Bengal rose (average removal 61.0%), and azo Congo red (average removal 57.4%). Obtained results revealed that the dye susceptibility to decolorization depends on the characteristic chemical structure of given dye groups but more important is chemical structure of strictly given dye within the group.

## Introduction

The high needs of nowdays consumers generate the common use of high amount of different synthetic dyes in all branches of economy. Popularity of these chemicals is connected with easy and low-cost production, very wide spectrum of colors, and durability of coloration. The most popular groups of dyes are azo and triphenylmethane ones. Their complicated, aromatic chemical structure causes their resistance to physical, chemical, and biological factors.

The dyeing processes always are associated with the loss of part of used dyes. These wastages depend on the property of dye used in the process and dyed material. In case of group of azo dyes, it is estimated that during dyeing process, about 15% of used dyes is lost (Chequer et al. 2011; Ghaly et al. 2014; Oliveira et al. 2007). It is assessed that even up to 40% of mass of all used dyes may be released to the wastewaters annually. Globally, only from the textile industry, 280,000 tons of different dyes may flow out with wastewater. It may be considered that the part of them may be illegal discharged to the surface waters (Ali 2010; Couto 2009; Ghaly et al. 2014).

The presence of dyes in wastewaters is more global then local problem. Dyes are released to the wastewaters both during their application (dyeing processes—more locally) and from using dyed products (e.g., releasing during washing textiles with weakly bounded dyes—widespread phenomenon) (Ali 2010; Couto 2009; Diwanian et al. 2010; Kalyani et al. 2009; Karatay et al. 2015; Lalnunhlimi and Krishnaswamy 2016; Leena and Selva 2008; Solis et al. 2012 Yadav et al. 2012). Technological problems and low efficiency of removal of dyes from sewage cause the introduction of dyes to the surface waters (with the cleaned sewages) and to the soils (with sewage sludges). These generate the serious environmental problems connected with disadvantageous changes of the physicochemical and biological properties especially in water ecosystems (Babu et al. 2007; Couto 2009; Forgacs et al. 2004; Karatay et al. 2015; Lalnunhlimi and Krishnaswamy 2016; Oranusi and Ogugbue 2005; Pearce et al. 2003; Rehman et al. 2015; Saratale et al. 2011). Resistance to physical, chemical, and biological factors generates a danger of accumulation of these substances in the environment. The presence of these xenobiotics in surface waters even in low concentration causes the changes of water color, which limits the light penetration and influences on limitation of primary production and may cause deficiency of oxygen (Kuhad et al. 2012; Lima et al. 2007; Mathur et al. 2005; Nese et al. 2007; Przystaś et al. 2012; Qadir and Chhipa 2015; Suryavathi et al. 2005; Verma and Mischra 2008; Zabłocka-Godlewska et al. 2014). The majority of the synthetic dyes may cause an acute toxicity and may be carcinogenic and/or mutagenic for organisms exposed to them. It is important to remember that these xenobiotics negatively impact on the biodiversity of water ecosystems (Ali 2010; Dawkar et al. 2009; Forgacs et al. 2004; Karatay et al. 2015; Li et al. 2015; Mendez-Paz et al. 2005; Oranusi and Ogugbue 2005; Paz et al. 2017; Pearce et al. 2003; Rauf and Ashraf 2012; Saratale et al. 2011; Solis et al. 2012; Ventura-Camargo and Marin-Morales 2013; Verma and Mischra 2008; Yamjala et al. 2016; Zabłocka-Godlewska et al. 2014; Zabłocka-Godlewska et al. 2015). The azo dyes, the group used at economy at the largest amounts, may affect adversely on organisms due to their carcinogenic, cytotoxic, genotoxic, mitotoxic, and also allergenic properties (Lima et al. 2007; Biswas and Khuda-Bukhsh 2005; Carita and Marin-Morales 2008; Tsuboy et al. 2007; Ventura-Camargo and Marin-Morales 2013). For example Mathur et al. (2005) revealed the mutagenicity of azo Congo red. Ventura-Camargo and Marin-Morales (2013) proved cytotoxic, genotoxic, mutagenic, and clastogenic properties of azo black commercial dye (BDCP) used for textile dyeing. Zabłocka-Godlewska et al. (2014, 2015) proved phytotoxicity and zootoxicity of azo Evans blue (concentration 0.08 g/l). Many dyes belonging to triphenylmethanes, another common group, proved their toxic, ecotoxic, carcenogenic, teratogenic, clastogenic, mitotoxic impact. Such properties are characterized for example as crystal violet, pararosaniline, malachite green, and brilliant green (Jang et al. 2004; Nandi and Patel 2013; Sharma et al. 2004; Wang et al. 2011; Zabłocka-Godlewska et al. (2014, 2015)).

Producing the large amount of dyed sewages and global character of this problem need the improvement of the used cleanup technologies. There is the need to increase their efficiency, cost-effectiveness, and environmental safety. Physical and chemical technologies are in general very efficient but are cost consuming and mostly connected with production of large amount of toxic sludges which are difficult to manage. Nowadays, the great interest is focused on the biological methods of decolorization. They are based on activity of bacteria and fungi, sometimes algae and plants. The important stage of developing of efficient biotechnological process is properly conducted a screening study. It allows to obtain organisms having capabilities of decolorization of wide spectrum of dyes with different chemical structures (Ali 2010; Forgacs et al. 2004; Holkar et al. 2014; Kuhad et al. 2012; Li et al. 2015; Mendez-Paz et al. 2005; Oranusi and Ogugbue 2005; Pandey et al. 2007; Paz et al. 2017; Pearce et al. 2003; Rauf and Ashraf 2012; Saratale et al. 2011; Solis et al. 2012; Srinivasan and Viraraghavan 2010).

The aim of presented studies was obtaining the new bacterial strains having a wide spectrum of decolorization of different synthetic dyes. Such properties are important for efficiency of cleaning up of dyed sewages due to their complex composition.

The bacterial strains were isolated from highly polluted liquid samples collected from two Upper Silesian (Poland) wastewater treatment plants and from highly polluted Silesian river Lesznica. In such polluted sites, the adaptation processes are expected which should influence on better capability of decolorization by bacteria living there.

The broad decolorization spectrum was studied with usage of different synthetic dyes belonging to three different chemical groups (azo, triphenylmethane, and fluorone dyes). Such choice of dyes considered the needs to study of the substances of various chemical structures.

## Materials and Methods

### Dyes Used in the Studies

The screening investigation was conducted with usage of six synthetic dyes from three different groups:

- Disazo dyes—Evans blue (EB), Congo red (CR) (Sigma-Aldrich)
- Triphenylmethane dyes—brilliant green (BG) (Sigma-Aldrich), crystal violet (CV) (POCH)
- Fluorone dyes—erythrosine (E), Bengal rose (BR) (Sigma-Aldrich)

The absorbance spectrum of each dye used in experiment was examined using spectrophotometer UV–Vis Hitachi 1900 (a wavelength range from 190 to 1100 nm). The maximum absorbance wavelength was determined (according to the single peak with the strongest absorption for each dye).

The structure, summary formulas and experimentally determined wavelengths corresponding to the maximal absorbance for used dyes are presented in Table 1.

**Table 1:**
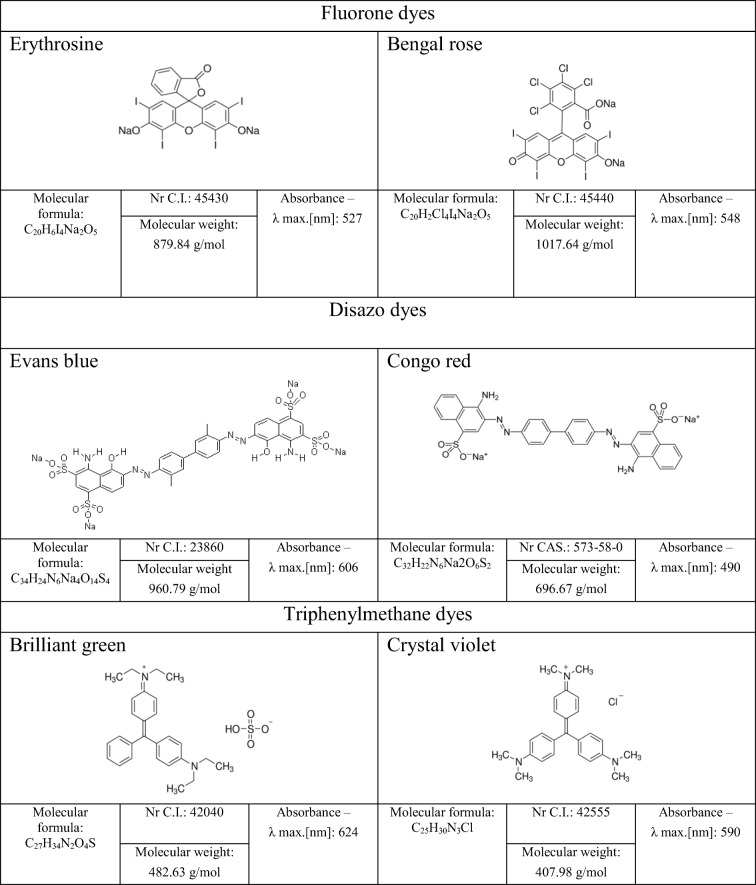
Dyes used in the study—characteristic (prepared mainly on the base on https://www.sigmaaldrich.com/poland.html)

The solutions of dyes were prepared in sterile distilled water. Each dye was dissolved in water and thoroughly mixed, and then, the solution was sterilized mechanically by cellulose syringe filters (pore diameter 0.2 μm). After sterilization, the real dye concentration in solution was measured by colorimetric method (spectrophotometer UV–Vis Hitachi 1900). Dye solutions were stored in the fridge in dark glass bottles until preparation of bacteriological growth media for bacterial cultivation. The concentrations of dyes were:

- 0.05 g/l and 0.1 g/l (BG or EB) in solid media—first stage of screening
- 0.1 g/l (all used dyes, each separately) in liquid media—second stage of screening

### Growth Media and Diluent

For cultivation of bacterial strains, the Kimura medium was used as solid and liquid form (ingredient contents per liter: 20 g glucose, 5 g peptone, 2 g yeast extract, 1 g KH_2_PO_4_, 0.5 g MgSO_4_x7H_2_O). The pH was adjusted on level 6.8–7.0. For preparation of solid media, bacteriological agar was used (18 g/l) (BTL).

Dilutions of samples (river water and sewage samples) and bacterial suspensions were prepared with usage of the physiological saline (0.85% water solution of NaCl).

The storage of bacterial strains and multiplication of bacterial biomass were done on the slants prepared from the nutrient agar (BTL).

### Isolation of Decolorizing Bacterial Strains

In the first stage of screening, solid Kimura medium supplemented with dyes (disazo Evans blue or triphenylmethane brilliant green) in concentration of 0.05 and 0.1 g/l was used. In this stage of studies, the strains of bacteria were isolated which had the ability to decolorize synthetic dyes.

The samples for isolation of bacteria were collected from aeration chambers of two municipal wastewater treatment plants in Silesia region in Poland (Świbie, Bytom) and highly polluted Silesian river Lesznica (Wodzisław Śląski). All samples were diluted in physiological saline (in range: 10^−1^–10^−6^—river water; 10^−1^–10^−9^—sewage samples). The volume 0.1 ml of every dilution was spread on plates with solid Kimura medium supplemented with suitable dye. Samples were incubated for 96 h in temperature 26 °C.

For further studies of decolorization potential, bacterial strains were chosen which grown with visible decolorized zone around the colony and/or had strongly dyed biomass. Based on these criteria, 67 bacterial strains were chosen with the ability to decolorize at least one of the used dyes. These strains were passaged onto the sterile media and were undergone to the purification procedure. The pure cultures of isolated bacteria were cultivated on agar slants in aim to multiply their biomass for further stage of screening.

### Studies of Decolorization Potential of Isolated Bacterial Strains

The studies of decolorization potential of isolated bacteria were conducted in tubes containing 9 ml of the liquid Kimura medium. Bacterial suspensions for sample inoculation were prepared from slants containing 48-h-age multiplied biomass of bacterial strains. They were prepared with use of physiological saline. The optical density of each suspension was adjusted to value ∼ 15 × 10^8^ cfu/ml. It was measured both with use of the McFarland Standards and spectrophotometric method at wavelength 600 nm. Each tube with 9 ml of the Kimura medium was inoculated with 0.1 ml of proper bacterial suspension. In aim to multiply the biomass, the samples were incubated in temperature 26 °C for about 48 h (up to reaching the stationary growth phase). The increase of biomass amount was monitored by measurements of optical density by spectrophotometric method at the wavelength of 600 nm.

After 48 h of incubation to each tube with multiplied bacterial biomass, the suitable sterile solution of dye (crystal violet, brilliant green, Evans blue, Congo red, erythrosine, Bengal rose) was introduced. The concentrations of dyes in liquid samples were 0.1 g/l. All samples were done in triplicate. The samples were incubated at 26 °C for 144 h. The control samples were prepared in the same way but without biomass. After incubation, all samples were centrifuged (5000 rpm/10 min) and then in the supernatants absorbance were measured (spectrophotometer UV–Vis Hitachi U-1900).

The maximal absorbance of used dyes was determined experimentally and is presented in Table 1. The percentage removal of each dye was calculated according to Formula 1.

1 $$ R=\left(C-S/C\right)\times 100\% $$

*R*: dye removal (%);
*C*: dye concentration in a control sample without biomass [mg/l];
*S*: dye residue concentration in a sample with bacterial biomass [mg/l].

### Initial Characterization of the Dye-Removing Bacterial Strains

Bacterial strains with the largest decolorization abilities were examined in aim to determine some of their properties such as the following: the result of Gram staining, morphology, motility, spore forming (microscopic analysis), producing of catalase, cytochrome c oxidase, the type of growth in liquid nutrient broth (determination if they are aerobic or facultative anaerobic bacteria).

Aerobic and anaerobic bacteria can be identified by the type of their growth in tube test with liquid nutrient broth.

The presence of catalase was detected by adding the drop of hydrogen peroxide to the drop of bacterial suspension placed on the microscope slide. The positive result was observed as release of oxygen bubbles.

Oxidase-positive bacterial strains (producing cytochrome c oxidase) were detected with the use of the oxidase reagent (N,N,N′,N′-tetramethyl-p-phenylenediamine dihydrochloride (Difco)). The oxidase reagent was applied directly on the bacterial biomass grown on nutrient agar slants. Oxidase-positive bacterial strains caused the change of color of reagent which is colorless in its reduced state and pink or dark purple in its oxidized state.

### Statistical Analysis

For statistical analysis, the program Statistica 5.1 (ANOVA/MANOVA test NIR) was used. To verify the assumed hypothesis, threshold of significance *p* < 0.05 was employed.

## Results and Discussion

Biological decolorization technologies offer an advantageous alternative of dyed sewage treatment. In comparison with the physical and chemical methods, they are relatively cheap and environmentally friendly technologies of removal of synthetic dyes from wastewater. Mentioned features cause the increasing interest of these biotechnologies. The general aim of studies at bioremediation of dyed effluents (e.g., textile effluents) is the improvement of degradation efficiency of the indigenous microorganisms. The bioaugmentation of indigenous microflora by properly selected microorganisms is a promising method that may allow improving the efficiency of degradation and mineralization of dyes with the low environmental impact and without using potentially toxic chemical substances (Karatay et al. 2015; Lalnunhlimi and Krishnaswamy 2016; Paz et al. 2017; Singh et al. 2015; Śekuljica et al. 2015; Yadav et al. 2012; Yamjala et al. 2016). In recent years, studies on development of biological decolorization technologies are focused among others on usage of a newly isolated microorganism (Asgher et al. 2014; Modi et al. 2010; Ougugbue et al. 2011; Paz et al. 2017). The aim of conducted screening investigation was the isolation of the new bacterial strains capable to decolorize various synthetic dyes. In highly polluted sites (e.g., textile industry effluents, municipal wastewaters, polluted surface waters, polluted soils and others), the presence of different complex substances caused that the microflora undergoes the specific selection and adaptation processes. Due to this, the contaminated sites may be consider a reach source of obtaining the microorganisms capable to the efficient decolorization of the synthetic dyes (Kandelbauer and Guebitz 2005; Khehra et al. 2005; Wang et al. 2011; Wu et al. 2009a; Zabłocka-Godlewska et al. 2012; Zabłocka-Godlewska et al. 2014; Zabłocka-Godlewska et al. 2015).

### Isolation of Decolorizing Bacterial Strains

The first stage of screening was conducted on the solid media supplemented with the suitable dyes (BG or EB). Decolorization abilities of the grown bacteria were estimated based on the occurrence of the decolorized zone around the colony and/or strongly dyed biomass. In this stage, 67 strains that exhibited such features were isolated and chosen for further examination.

The larger number of bacterial colonies that exhibited dyed biomass and decolorization zones was counted on the plates with BG than with EB. From samples supplemented with triphenylmethane BG, 46 bacterial strains were isolated, and from samples with azo EB, only 21 strains were isolated. Most of the isolates were obtained from samples collected from WTP Bytom (35 strains, 14 EB and 21 BG), then from WTP Świbie 12 (3 EB and 9 BG) and from river water Lesznica (20 isolates—4 EB and 16 BG). These preliminary results showed that the differences of chemical structure of used dyes may influence on their decolorization possibilities by direct impact on bacteria and different bioavailabilities as a substrate. Triphenylmethane brilliant green was the substrate easier for assimilation than disazo Evans blue which was proven in other studies (Przystaś et al. 2009; Zabłocka-Godlewska et al. 2014; Zabłocka-Godlewska et al. 2015).

Bacteria with dyed biomass prevailed over the colorless strains with a presence of the decolorization zone. It suggests that sorption processes may play an important role in decolorization processes, and even, it may be the main mechanism of dye removal from liquid solutions (Wang et al. 2011; Wu et al. 2009a; Wu et al. 2009b; Zabłocka-Godlewska et al. 2015). The most of bacterial colonies which adsorbed and/or decolorize medium were detected in samples from WTP Bytom (WTPB), then from WTP Świbie (WTPS) and river Lesznica (RL). Their average participation [%] in the total bacterial number [cfu] was as follows: WTPB—in case of BG 78.4%, EB 64.1%; WTPS—BG 72.6%, EB 58.3%; RL—BG 62.1%, EB 42.4%. In decolorizing bacterial number, the participation of strains with decolorized zones was ranged between 37.2 and 44.6% in case of BG and between 24.1 and 32.9% in case of EB.

### Bacterial Decolorization Potential

**Fig. 1:**
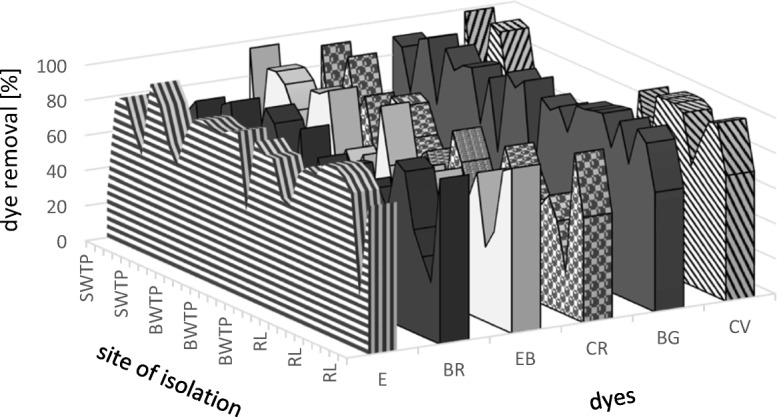
Percentage removal of different dyes by bacterial strains isolated from different environments (SWTP, Świbie water treatment plant; BWTP, Bytom water treatment plant; RL, river Lesznica. Dyes: E, erythrosine; BR, Bengal rose; EB, Evans blue; CR, Congo red; BG, brilliant green; CV, crystal violet)

Studies conducted in the second stage showed substantial differences in decolorization potential of isolated bacterial strains. Less than 50% from them (31 from 67) were capable to decolorize with different efficiencies all of dyes used in studies (Fig. 1; Table 2). Decolorization efficiency depended on the group of studied dyes which was connected with characteristic chemical structure of groups and differences between them. This dependence was well documented also in other studies (An et al. 2002; Chander and Arora 2007; Jang et al. 2005; Sani and Banerjee 1999; Novotny et al. 2004; Przystaś et al. 2009; Zabłocka-Godlewska et al. 2014; Zabłocka-Godlewska et al. 2015). The best removal results were reached for triphenylmethane and fluorone dyes, and azo dyes were removed with lower efficiency, which confirms the results of preliminary studies (the first stage of screening). Average percentage removal effectiveness was 75.6, 70.0, and 60.9% respectively.

**Table 2:** Percentage removal of the dyes in liquid samples after 6 days of incubation (italicized values—samples with dye removal ≥ 75%)

Samples for bacterial isolation collected from:	Bacterial strains^2^	Percentage removal of dyes after 6 days of incubation (initial concentration 0.1 g/l)					
		Fluorone	Azo			Triphenylmethane	
		Erythrosine	Bengal rose	Evans blue	Congo red	Brilliant green	Crystal violet
WTP^1^ Świbie—aeration tank	Sb1	24.0 ± 0.1	30.1 ± 0.1	*95.7 ± 0.4*	*91.1 ± 2.2*	*90.1 ± 0.1*	*98.4 ± 0.2*
	Sz1	*81.2 ± 2.1*	67.9 ± 2.9	31.1 ± 1.0	44.0 ± 0.7	*86.5 ± 0.4*	56.4 ± 0.7
	Sz2	*84.3 ± 1.9*	*76.0 ± 0.6*	*88.5 ± 0.8*	62.2 ± 1.8	62.7 ± 0.4	56.6 ± 2.8
	Sz3	67.5 ± 2.4	28.3 ± 0.2	*89.0 ± 1.0*	*91.2 ± 1.4*	*95.5 ± 0.5*	*98.2 ± 0.6*
	Sz4	55.1 ± 0.2	23.6 ± 1.0	*83.9 ± 0.5*	59.8 ± 2.5	*98.2 ± 0.1*	*95.7 ± 0.2*
	ARDG*	**62.42**	**45.18**	***77.64***	**69.66**	***86.60***	***81.06***
	ARG**	**53.8**		**73.65**		***83.83***	
WTP^1^ Bytom—aeration tank	Bb1	*98.5 ± 0.6*	*80.6 ± 0.6*	71.1 ± 1.1	73.3 ± 0.5	62.7 ± 1.4	62.8 ± 0.9
	Bb2	*88.1 ± 0.1*	*84.4 ± 0.7*	44.2 ± 0.1	44.9 ± 1.3	*96.7 ± 0.8*	65.3 ± 3.1
	Bb3	68.2 ± 0.2	33.3 ± 0.9	*87.2 ± 0.9*	49.8 ± 0.4	*86.4 ± 0.5*	57.7 ± 0.5
	Bb4	57.8 ± 0.4	30.3 ± 1.3	*87.5 ± 1.0*	*79.5 ± 0.9*	*91.5 ± 0.6*	59.6 ± 0.4
	Bb5	*77.7 ± 0.4*	64.2 ± 0.6	*91.4 ± 0.9*	*76.4 ± 1.1*	*92.4 ± 0.2*	57.6 ± 0.5
	Bb6	*89.5 ± 0.5*	*89.7 ± 0.3*	46.2 ± 0.9	60.2 ± 2.0	62.7 ± 1.0	50.6 ± 0.4
	Bb7	*91.0 ± 1.1*	*83.2 ± 0.7*	62.2 ± 0.4	50.1 ± 0.4	*91.0 ± 0.0*	31.1 ± 0.7
	Bb9	*88.7 ± 2.8*	64.4 ± 0.4	50.4 ± 0.9	57.4 ± 1.1	51.0 ± 0.9	64.9 ± 1.0
	Bz1	*87.7 ± 1.4*	34.1 ± 1.1	63.6 ± 0.6	43.8 ± 1.6	*98.5 ± 0.5*	13.4 ± 0.8
	Bz2	*91.5 ± 0.9*	*86.7 ± 1.0*	28.7 ± 0.6	27.4 ± 0.2	*92.1 ± 0.2*	35.6 ± 0.9
	Bz3	*93.5 ± 0.4*	10.4 ± 0.1	*95.9 ± 0.2*	75.7 ± 1.2	*98.1 ± 0.2*	56.9 ± 0.4
	Bz5	50.2 ± 0.9	74.7 ± 0.4	64.1 ± 0.5	60.0 ± 1.3	32.3 ± 0.5	24.4 ± 0.2
	Bz6	*92.1 ± 0.7*	56.6 ± 1.2	37.8 ± 1.0	41.3 ± 1.0	*92.9 ± 0.4*	30.9 ± 0.4
	Bz7	*86.7 ± 1.2*	*75.3 ± 0.5*	22.2 ± 0.6	29.1 ± 0.3	*88.2 ± 0.3*	13.3 ± 0.7
	Bz8	*86.8 ± 0.4*	*76.5 ± 0.2*	69.4 ± 0.6	31.4 ± 0.9	*92.3 ± 0.3*	31.5 ± 0.3
	ARDG*	***83.2***	**62.96**	**61.46**	**53.35**	***81.92***	**43.71**
	ARG**	**73.08**		**57.41**		**62.82**	
Lesznica River Wodzisław Śląski	Rb1	66.6 ± 1.8	46.4 ± 1.6	39.9 ± 0.9	52.3 ± 0.6	*80.0 ± 0.1*	*97.4 ± 0.3*
	Rb2	63.6 ± 4.4	72.4 ± 0.8	71.8 ± 0.7	*80.1 ± 2.1*	*97.2 ± 0.2*	58.6 ± 0.5
	Rb3	*84.8 ± 0.6*	52.2 ± 1.2	42.9 ± 1.5	64.2 ± 0.5	*97.4 ± 0.1*	*99.0 ± 0.2*
	Rb4	*90.9 ± 0.0*	73.4 ± 0.3	59.8 ± 0.9	53.0 ± 1.1	*98.8 ± 0.7*	*99.4 ± 0.0*
	Rz1	*88.5 ± 0.8*	63.4 ± 0.7	44.0 ± 1.1	32.2 ± 0.7	*99.6 ± 0.1*	*99.2 ± 0.2*
	Rz2	*97.1 ± 0.3*	*92.9 ± 0.7*	65.7 ± 0.7	55.1 ± 0.9	*82.3 ± 0.5*	*96.0 ± 2.0*
	Rz3	*97.9 ± 0.6*	*89.0 ± 0.4*	*82.4 ± 0.6*	63.5 ± 0.6	*99.5 ± 0.0*	68.3 ± 0.9
	Rz4	*94.8 ± 0.4*	58.4 ± 1.6	42.4 ± 0.7	51.1 ± 0.7	*77.5 ± 0.4*	*84.0 ± 0.3*
	Rz5	*87.5 ± 0.9*	45.1 ± 2.8	53.5 ± 1.4	23.9 ± 1.7	*99.4 ± 0.4*	*97.4 ± 0.2*
	Rz6	29.3 ± 0.4	32.9 ± 1.4	*90.1 ± 0.4*	*94.0 ± 2.4*	*93.7 ± 0.1*	*99.3 ± 0.1*
	Rz8	*84.5 ± 0.5*	*93.8 ± 1.3*	*93.7 ± 0.8*	59.9 ± 3.8	68.1 ± 0.2	71.4 ± 0.5
	ARDG*	***80.5***	**65.45**	**62.38**	**57.21**	***90.32***	***88.18***
	ARG**	**72.98**		**60.9**		***75.6***	
Average removal of dyes [%]		***78.9***	**61.0**	**64.4**	**57.4**	***85.7***	**65.5**
Average removal of group of dyes [%]		**70.0**		**60.9**		***75.6***	

Bolded and italicized were values of dyes removal which were on level > or = 75%

^1^*WTP*—wastewater treatment plant

^2^Bacterial strain description—Aax: where A—the site of collection sample for isolation; a = z—it means strain isolated from plates with brilliant green; a = b—it means strain isolated from plates with Evans blue; x—the number of strain

*ARDG—average removal of dye in given group

**ARG—average removal of given group of dyes

All liquid samples for isolation of bacteria were collected from highly polluted sites. That choice was dictated by the question if there are the possibilities of obtaining from these sites the microorganisms able to decolorize the synthetic dyes efficiently. In such environments, the processes of selection and adaptation promote the development of microorganisms which are resistant to different negative factors and are capable to degrade the substances even low biodegradable. Such properties are very important in case of difficult for treatment industry sewages, e.g., textile effluents. The municipal wastewaters are constantly enriched with synthetic dyes among others by discharge of the effluents generated during clothes washing. During the washing operations, the textiles that are dyed may release to the water the mixture of synthetic dyes. The polluted surface waters also may include the synthetic dyes especially in highly industrial areas. It should be considered the possibilities of illegal discharge the sewages from households and from small factories (textile, tanneries). Considering the above, we may expect that chosen polluted environments may be a good source of isolation of required microorganisms.

The differences of composition of contaminants between environments cause the differences of the degradation potential of the microorganisms. Results of conducted studies showed some differences between decolorization efficiency of strains isolated from given sites (Fig. 1). The efficiency of decolorization of fluorone group (erythrosine, Bengal rose) was the lowest in case of strains from WTP Świbie (average removal 53.8%). The higher removal efficiency of fluorone dyes was detected in case of strains from WTP Bytom and from river Lesznica (average removal 73.08 and 72.98% respectively). The best efficiency of removal of azo and triphenylmethane groups was reached in case of strains isolated from WTP Świbie (average removal 73.65 and 83.83% respectively), followed by the strains from river Lesznica and WTP Bytom (average removal 57.41 and 62.82% and 60.9 and 75.6% respectively).

Significant differences of decolorization abilities by strains were also observed within the specific group of dyes. The differences in the structure of dyes belonging to the same group strongly determined the efficiency of their removal (Fig. 1; Table 2). In fluorone group, Bengal rose having more complicated chemical structure with chlorine substituents was decolorized with significantly lower efficiency than structurally simpler erythrosine (dye removal ≥ 75% was reached respectively by 12 and 22 strains from 31 studied). Average percentage removal effectiveness was 78.9 and 61.0% respectively. These results confirm the results of our previous studies conducted with the use of other 61 bacterial strains (Przystaś et al. 2009; Zabłocka-Godlewska et al. 2016).

The next studied group was azo dyes. Evans blue and Congo red are disazo dyes. The azo dyes which have in the structure the electronoacceptors groups (such SO_3_H, SO_2_NH_2_, COOH) are more biodegradable than those in electronodonative groups such as NH_2_ and OH groups. In comparison with Congo red the Evans blue has got in molecule more electroacceptors groups, what probably was the reason of its higher removal results than in case of Congo red (dye removal ≥ 75% was reached respectively by 11 and 6 strains from 31 of studied). The average percentage of removal efficiency of these dyes was respectively 64.4 and 57.4%. The same tendency was observed in our previous studies (Zabłocka-Godlewska et al. 2016).

The last examined group of dyes was triphenylmethanes (brilliant green and crystal violet). Much higher efficiency of decolorization was observed in case of brilliant green which has simpler chemical structure (two ethyl groups connected with two phenyl rings) than crystal violet (three ethyl groups connected with three phenyl rings). The efficiency of brilliant green removal ≥ 75% was reached by 25 from 31 studied strains, when crystal violet was removed on such level only by 11 strains (Fig. 1, Table 2). The average percentage removal was respectively 85.7 and 65.5%. This tendency was also observed in our previous studies with another 61 bacterial strains isolated from different sites (Przystaś et al. 2009; Zabłocka-Godlewska et al. 2016).

The comparison of the results of decolorization of all used dyes allows ordering them according to their susceptibility to the biotransformation (from the lowest to the highest removal efficiency; in brackets are presented the values of average percentage of the dye removal):

$$ \mathrm{CR}\ \left(57.4\%\right)<\mathrm{BR}\ \left(61.0\%\right)<\mathrm{E}\mathrm{B}\ \left(64.4\%\right)<\mathrm{CV}\ \left(65.5\%\right)<\mathrm{E}\ \left(78.9\%\right)<\mathrm{BG}\ \left(85.7\%\right) $$

Arrangement of these dyes according to the number of strains that reached the removal level ≥ 75% looks similar (in brackets is the number of strains that exhibited such high efficiency of the dye removal):

$$ \mathrm{CR}\ (6)<\mathrm{CV}\ \mathrm{and}\ \mathrm{EB}\ (11)<\mathrm{BR}\ (12)<\mathrm{E}\ (22)<\mathrm{BG}\ (25). $$

There were no statistically significant differences (ANOVA/MANOVA test NIR) between the sites of origin of isolated bacteria and their ability to decolorize the given chemical group of dyes (fluorone, azo, and triphenylmethane). The same was demonstrated in case of analysis of statistically differences between the isolation sites of bacteria and their ability to decolorize the given dye. The exception was the crystal violet. ANOVA/MANOVA analysis demonstrated statistically significant differences (*p* < 0.05) of the results of crystal violet removal between the bacterial strains isolated from WTP Bytom and isolates from WTP Świbie and also between bacteria from WTP Bytom and river Lesznica.

The comparison of the results of decolorization of the studied dyes by bacteria isolated from given source was done. For strains isolated from WTP Świbie, the statistically significant differences (*p* < 0.05) were demonstrated between the results of removal of Bengal rose and the rest of the dyes (with exception of Congo red) and between decolorization results of fluorone and triphenylmethane groups. In case of strains isolated from WTP Bytom, the statistically significant differences (*p* < 0.05) were demonstrated between the abilities of the Bengal rose decolorization and erythrosine and brilliant green and also between the results for crystal violet and erythrosine and brilliant green. Statistically significant differences were also demonstrated for decolorization results of fluorone and azo groups and between azo and triphenylmethane groups. For strains isolated from Lesznica river, the statistically significant differences (*p* < 0.05) were demonstrated only between the abilities of Congo red decolorization and brilliant green, crystal violet, and between azo and triphenylmethane groups.

The comparison of the decolorization results of the studied dyes showed the specific abilities of microflora isolated from the given polluted site. However, the statistical analysis demonstrated that differences between the decolorization potential of bacteria isolated from different sites were slight.

### Initial Characterization of the Dye-Removing Bacterial Strains

Initial characterization of examined bacteria (31 strains) included estimation of some morphological and physiological features (Table 3). The growth typical for aerobic bacteria (the film on the surface of liquid Kimura medium) was exhibited by 32% of strains while 78% of bacterial strain grown in way typical for facultative anaerobes (occurrence of turbidity of medium). The Gram staining shows the differences in the cell wall structure of isolated bacterial strains: 21% of bacterial strains were Gram positive while 79% were Gram negative. Microscopic analyses showed that 77% of isolated bacteria were rod shaped, and the rest (23%) were streptobacilli, streptococci, or staphylcocci form. The majority of strains were catalase positive (84%) and oxidase negative (68%). Only 13% of strains had the abilities to form spores. The motility was detected in case of 52% of them. The results of initial characterization of examined decolorizing bacteria are similar to the results reached by other researchers. The most of studied strains are Gram negative, rod shaped, facultative anaerobes (Ali 2010; Santos et al. 2007; Saratale et al. 2011; Solis et al. 2012; Srinivasan and Viraraghavan 2010). The results presented above may suggest that there are correlations between the studied features and decolorization potential. However, the ANOVA/MANOVA analysis showed that there were no statistically significant differences between the specific studied features of bacteria and their decolorization potential (neither in case of a single dye nor the chemical group of dyes). The exception was triphenylmethane crystal violet. In case of this dye, the efficiency of removal was connected with the type of respiration of bacteria. The most effective decolorization was reached by facultative anaerobes than in case of aerobes (there were statistically significant differences between results of dye removal (*p* < 0.05)).

**Table 3:** Initial characterization of the dye-removing bacterial strains

Samples for bacterial isolation collected from:	Bacterial strain	Selected characteristics of bacteria						Selected characteristics of bacteria
		Result of Gram staining	Morphology (cell shape)	Motility	Spores formulation	Catalase producing	Cytochrome c oxidase producing	Type of growth in liquid medium
WTP^1^ Świbie—aeration tank	Sb1	–	Rod shaped	–	–	+	–	Facultative anaerobes
	Sz1	–	Rod shaped	+	–	+	+	Aerobes
	Sz2	+	Streptobacilli	+	+	+	+	Aerobes
	Sz3	–	Rod shaped	+	–	+	–	Facultative anaerobes
	Sz4	–	Rod shaped	–	–	+	–	Facultative anaerobes
WTP^1^ Bytom—aeration tank	Bb1	–	Rod shaped	+	–	+	–	Facultative anaerobes
	Bb2	–	Rod shaped	+	–	+	–	Facultative anaerobes
	Bb3	±	Rod shaped	–	–	+	+	Aerobes
	Bb4	+	Staphylococci	–	–	+	–	Facultative anaerobes
	Bb5	–	Rod shaped	+	–	+	+	Aerobes
	Bb6	–	Rod shaped	+	–	+	–	Facultative anaerobes
	Bb7	–	Rod shaped	+	–	+	+	Aerobes
	Bb9	+	Streptococci	–	–	–	–	Facultative anaerobes
	Bz1	–	Rod shaped	+	–	+	+	Aerobes
	Bz2	–	Rod shaped	–	–	+	–	Facultative anaerobes
	Bz3	+	Streptobacilli	–	+	–	–	Facultative anaerobes
	Bz5	–	Rod shaped	–	–	+	–	Facultative anaerobes
	Bz6	+	Streptobacilli	+	+	+	+	Aerobes
	Bz7	–	Rod shaped	–	–	–	–	Facultative anaerobes
	Bz8	–	Rod shaped	+	–	+	–	Facultative anaerobes
Lesznica River Wodzisław Śląski	Rb1	–	Rod shaped	–	–	–	–	Facultative anaerobes
	Rb2	+	Rod shaped	+	–	+	–	Facultative anaerobes
	Rb3	–	Rod shaped	+	–	+	+	Aerobes
	Rb4	–	Rod shaped	–	–	+	–	Facultative anaerobes
	Rz1	+	Streptococci	–	–	–	–	Facultative anaerobes
	Rz2	–	Rod shaped	+	–	+	–	Facultative anaerobes
	Rz3	+	Streptobacilli	–	+	+	–	Facultative anaerobes
	Rz4	–	Rod shaped	+	–	+	–	Facultative anaerobes
	Rz5	–	Rod shaped	–	–	+	+	Aerobes
	Rz6	–	Rod shaped	–	–	–	–	Facultative anaerobes
	Rz8	–	Rod shaped	+	–	+	+	Aerobes

## Conclusions

The conducted screening showed that apart from industrial wastewater, municipal sewages as well as water from polluted river may be the precious sources of strains of bacteria having the wide decolorization potential. Among 67 strains, 31 had the ability to remove of all studied dyes belonging to three different chemical groups. The effectiveness of decolorization was different and mainly depended on the structure of the dye. The comparison of decolorization results of all used dyes allowed ordering them according to their susceptibility to removal (from the highest to the lowest result of removal) in the order: brilliant green, erythrosine, crystal violet, Evans blue, Bengal rose, and Congo red. The dye group that was removed with the highest yield was triphenylmethane, followed by fluorone and azo. The majority of decolorizing bacteria were Gram negative, rod shaped, facultative anaerobes, catalase negative, and oxidase negative.
